# Impairement of HT29 Cancer Cells Cohesion by the Soluble Form of Neurotensin Receptor-3

**DOI:** 10.18632/genesandcancer.22

**Published:** 2014-07

**Authors:** Fabienne Massa, Christelle Devader, Sandra Lacas-Gervais, Sophie Béraud-Dufour, Thierry Coppola, Jean Mazella

**Affiliations:** ^1^ Institut de Pharmacologie Moléculaire et Cellulaire, Université de Nice-Sophia Antipolis, Valbonne, France.; ^2^ Centre Commun de Microscopie Appliquée, Université de Nice-Sophia Antipolis, Nice, France

**Keywords:** soluble sortilin, cell morphology, desmosomes, cancer, neurotensin

## Abstract

The neurotensin (NT) receptor-3 (NTSR3), also called sortilin is a multifunctional protein localized at the intracellular and plasma membrane level. The extracellular domain of NTSR3 (sNTSR3) is released by shedding from several cell lines including colonic cancer cells. This soluble protein acts as an active ligand through its ability to bind, to be internalized in the human adenocarcinoma epithelial HT29 cells and to stimulate the PI3 kinase pathway. The aim of this study was to investigate cellular responses induced by sNTSR3 in HT29 cells. The cellular functions of sNTSR3 were monitored by immunofluocytochemistry, electron microscopy and quantitative PCR in order to characterize the cell shape and the expression of adhesion proteins. We evidenced that sNTSR3 significantly regulates the cellular morphology as well as the cell-cell and the cell-matrix adherens properties by decreasing the expession of several integrins and by modifying the structure of desmosomes. Altogether, these properties lead to an increase of cell detachment upon sNTSR3 treatment on HT29, HCT116 and SW620 cancer cells. Our results indicate that sNTSR3 may induce the first phase of a process which weaken HT29 epithelial properties including desmosome architecture, cell spreading, and initiation of cell separation, all events which could be responsible for cancer metastasis.

## INTRODUCTION

Numerous extracellular molecules are involved in the activation of gastrointestinal cancer tissues growth. Among these effectors, several neuropeptides are known to act as growth factors through their receptors which are often overexpressed in tumors [1]. These neuropeptidergic systems are targeted for diagnosis and therapy by tools designed to modulate receptors activities [2]. From these tumors, some cells can be disseminated by complex mechanisms leading to the appearance of metastasis [3].

Molecules that are responsible for the development of both cancer growth and metastasis are either abnormaly secreted from intracellular secretion vesicles like neuropeptides or released by shedding from the plasma membrane like Epidermal Growth Factor Receptor (EGFR) ligands. Indeed, EGFR ligands are transmembrane proteins that can be cleaved by matrix metalloproteases (MMPs) and released in the extracellular medium to act on their receptors [4]. Another family of proteins has been recently shown to be shedded by similar mechanisms. This family, named Vps10p protein family [5], is constituted by type I receptor proteins and includes sortilin, SorLa, and SorCs 1-4 [6]. The extracellular part of these proteins can be released from the plasma membrane by MMPs [7,8]. However, the putative function of these soluble proteins, as well as the action of their counterpart intracellular domains, remain to be determined. The roles of the proteins from the Vps10p family are already known to be multiple and complex, and concern their functions as receptors or co-receptors and their involvement in the sorting of proteins to lysosomes or to the plasma membrane (for review, see [9]).

Recently, the soluble form of sortilin [10], also called neurotensin (NT) receptor-3 (NTSR3) [11], has been described as a functional molecule for its ability to bind and to be internalized in the human adenocarcinoma cell line HT29 [12]. In this work, it has been also demonstrated that sNTSR3 activated both Akt and Erk1/2 signaling pathways by a mechanism dependent on the focal adhesion kinase.

In the present study, we sought to examine the role of sNTSR3 on the morphology and behaviour of the colonic epithelial cancer cells HT29. Our data show that sNTSR3 incubation on HT29 cells leads to several changes in cell shape including actin reorganization and cell morphology in relation to a decrease of several integrins. sNTSR3 also induces a strong modification in the architecture of desmosomes. We demonstrate that sNTSR3 induces detachment of several colonic cancer cells including HT29, HCT116 and SW620 cell lines. Taken together, our results demonstrate that the soluble form of NTSR3 may regulate, by a complex mechanism, the fate of the human colonic adenocarcinoma cell line HT29, especially during the initial step of cell detachment.

## RESULTS

### Morphological changes of HT29 cells induced by sNTSR3

We previously demonstrated that sNTR3 activates the PI3 kinase pathway and increases the intracellular concentration of calcium [12]. Since these pathways play significant roles in cell morphology [14,15], we attempted to evaluate the role of sNTSR3 in the fate of HT29 cells.

**Figure 1:**
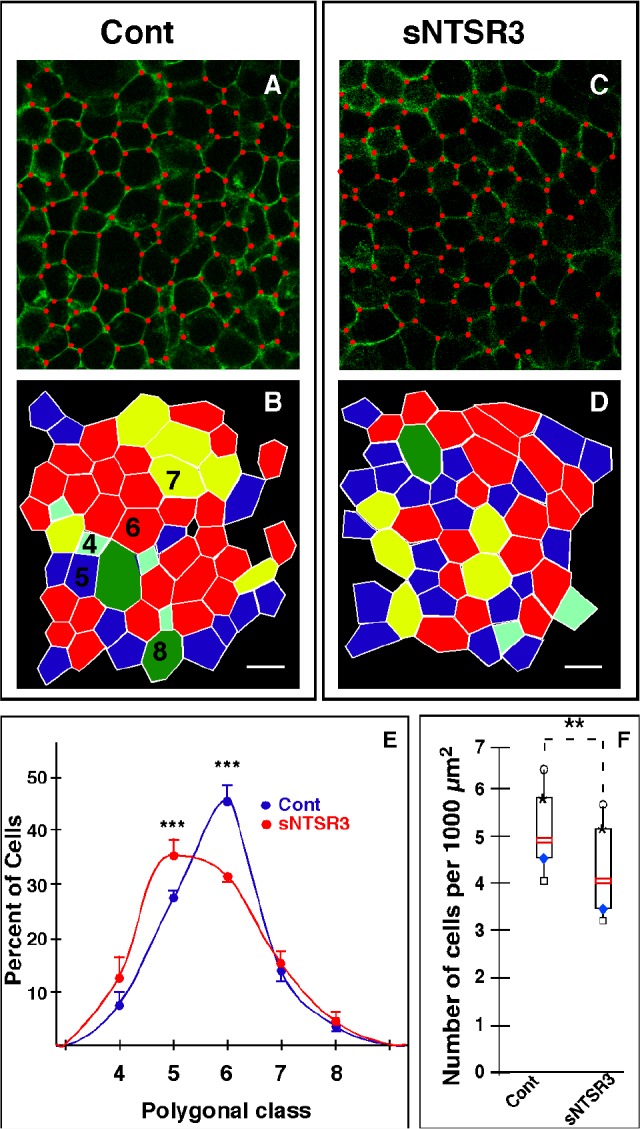
sNTSR3 regulates HT29 cell topology A-D- Cells were serum-starved and stimulated (C) or not (A) with sNTSR3 (10^−8^M) for 15h. Vertices were identified with red dots to count the number of edges of each cell. B and D assigned polygon classes of A and C respectively, tetragons (cyan), pentagons (blue), hexagons (red), heptagons (yellow) and octagons (green). E- Distribution of polygon classes for control (blue circles) or following sNTSR3 incubation (red circles). Scale bar = 20 μm; values are mean ± SEM from three independent determinations (*** p<0.005). The number of counted cells was 252 for resting cells and 265 for sNTSR3 treated cells from 3 independent experiments. F- Cells were numbered by image analysis using ImageJ (Wayne Rasband, National Institute of Mental Health, Bethesda, Maryland, USA) software and an home-made macro to detect and count nuclei normalized by a surface unit for each image. The number of cells per 1000 μm^2^ was expressed as median value obtained from 10 determinations for each condition, box plots show medians, 25% and 75% percentiles, and 5% and 95% percentiles. **p < 0.01. The number of counted cells was 520 for resting cells and 372 for sNTSR3 treateed cells from 5 independent experiments.

Using confocal imaging, we observed that the geometric cell shape of HT29 cells, analyzed after E-cadherin labeling, was modified after sNTSR3 treatment. The geometric distribution (polygon classes) of epithelial mammalian cells has recently been described to reflect their modifications of states [16]. In resting cells, the geometric distribution showed that 46% of HT29 cells were hexagons (Fig. 1A, B, E) consistent with published data on keratinocytes [16] and in drosophila epithelial cells [17]. Interestingly, in sNTSR3 treated cells, we observed a significant reduction in the proportion of hexagons (p<0,01), Fig. 1E) with a concomitant increase in the proportion of pentagons detected after 60 min and maintained up to 15 h (p<0,01, Fig. 1C, D, E). Moreover, in cells incubated in the presence of sNTSR3 the alteration of cell topology was associated with an increase in cell surface. The size of HT29 cells was assessed by counting the number of cells per 1000 μm^2^ which varied from 5.1 ± 0.2 cells in the absence of sNTSR3 to 4.2 ± 0.17 cells in the presence of 10 nM sNTSR3 for 30 min (Fig. 1F) (n = 25, p<0.01). This significant decrease in the number of cells per mm^2^ suggests an increase of the cell surface.

**Figure 2:**
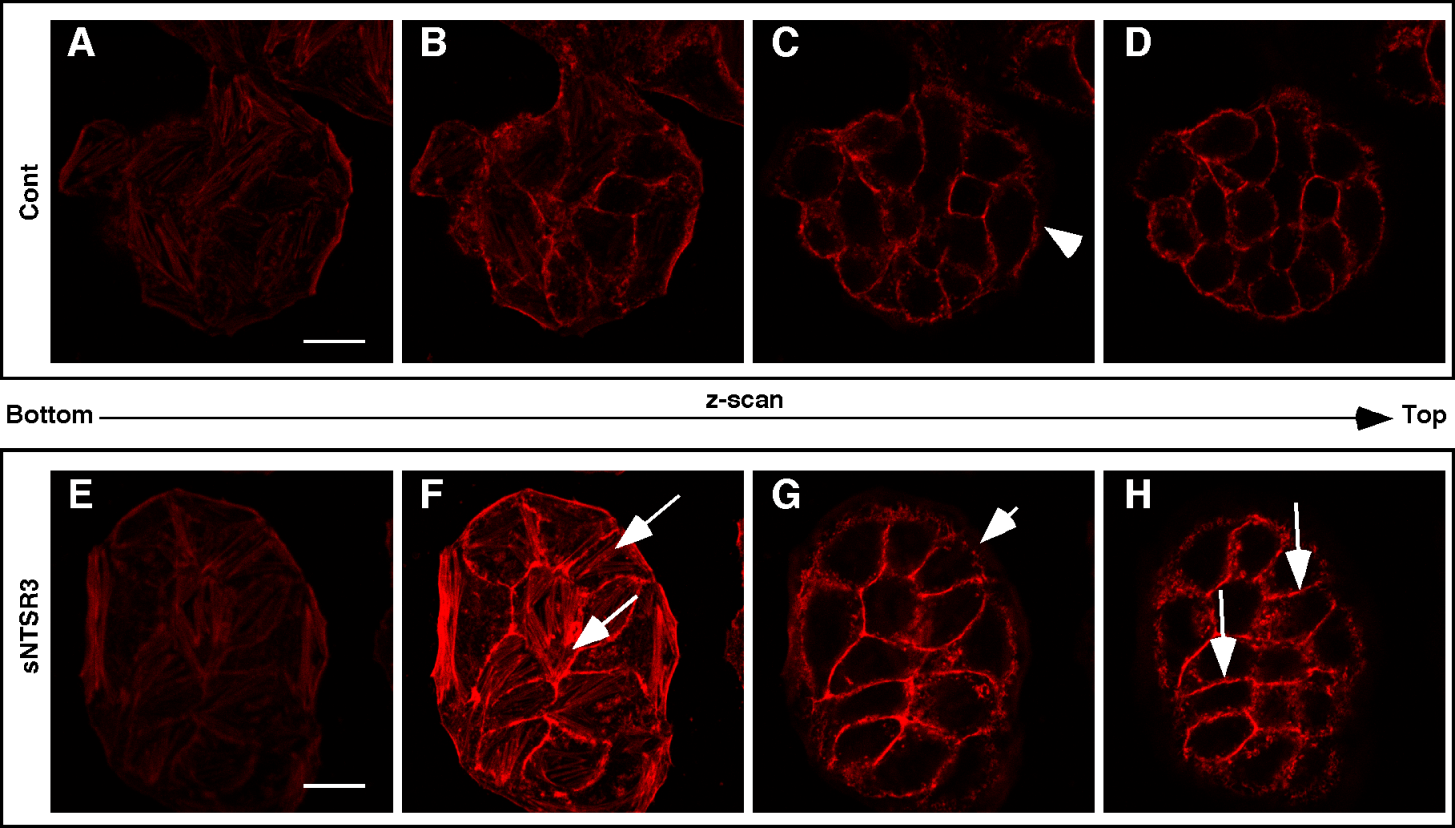
Morphological and biophysical changes of sNTSR3-stimulated HT29 cells Cells were serum-starved and incubated in the absence (A-D) or in the presence (E-H) of sNTSR3 (10^−8^M) for 15 min. Actin cytoskeleton was visualized using actin Texas-Red Phalloidin and series of z-scan were made. Arrows show actin stress fiber formation. Arrowheads indicated a disruption of actin labeling throughout the membrane of peripherical cells upon stimulation with sNTSR3 compared to non-treated cells. Fine arrows point out an increase of actin concentration in cell junctions (Fig. 2H). Scal bar : 10 μm. This experiment was representative from 3 independent experiments.

These observations led us to study the cytoskeleton modification induced by sNTSR3 treatment. Therefore, we examined the shape of actin cytoskeleton upon stimulation with sNTSR3. From a series of a z-scan performed by confocal microscopy from the bottom to the top of cell clusters, we observed several important changes on the cell morphology. First of all, we visualized an increase of actin stress fibers (Fig. 2F, arrows) and a disruption of actin labeling throughout the membrane of peripherical cells (arrowheads) upon stimulation with 10 nM sNTSR3 (Fig. 2G) compared to non-treated cells (Fig. 2C). Interestingly, we also observed an increase of actin concentration in cell junctions (Fig. 2H, fine arrows).

**Figure 3:**
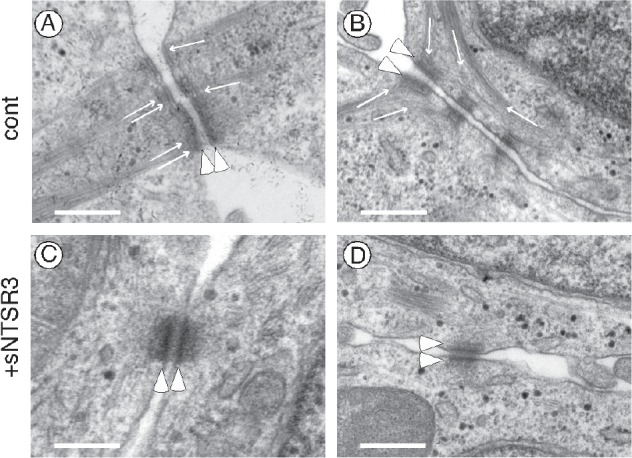
Electron microscopy of HT29 cells (A-B) Electron microscopy observation of resting cells showed numerous well structured desmosomes at the cell-cell contacts visualized by electron-dense plaques (arrowheads). Intermediate filaments were indicated by thin arrows. In many control cells, intermediate filament bundles formed right angles to the plaque densities (A) or were strongly linked to the vicinity of plaques (A and B). (C-D) In cells treated with sNTSR3 for 60 min, the architecture of desmosomes was often disrupted with either the absence of intermediate filaments (C) or the presence of a small number of intermediate filament or ambigous density near the plaque (D). Scale bars correspond to 500 nm. These results were representative from images taken from 4 independent experiments.

In agreement with a reorganization of actin microfilaments and a change of cell shape, we wanted to determine whether some ultrastructural components were altered. Using electron microscopy, we observed in sNTSR3 treated cells a modification in the architecture of numerous desmosomes and intermediate filaments (Fig. 3). Desmosomes fortify cell-cell adhesion by connecting proteins forming these structures to the intermediate filament cytoskeleton and therefore participate to tissue integrity and homeostasis [18]. From a series of electron microscopic images taken under control or sNTSR3 stimulated HT29 cells conditions, we counted the average number of desmosomes per 70 nm cell slice. A decrease from 5.06±0.34 desmosomes/cell slice (189 desmosomes counted) in control to 3.63±0.31 (p<0.01) desmosomes/cell slice (156 desmosomes counted) in treated cells was quantified (Table 2). More important was the observation that, although intercellular densities associated with cadherins appeared to be similar in both conditions, sNTSR3 treatment caused distinct changes in desmosomal architecture (Fig. 3). The plaque densities are generally associated with intermediate filament bundles in the resting cells (Fig. 3A and B), this was not the case for sNTSR3 treated cells where intermediate filament bundles were rarely visible in the close vicinity of desmosomes (Fig. 3C and D). In numerous resting cells, the intermediate filament bundles were strongly observable. Some intermediate filaments were arranged at right angles to the plane of desmosomes (Fig. 3A), others were more tangential (Fig. 3B). By contrast many sNTSR3 treated cells showed plaque densities without or with weak intermediate filaments (Fig. 3C and D). Therefore, we scored (from 0 to 3) all desmosomes obtained in the two conditions [19]. The results (Table 2) indicated an important loss of intermediate filament connections (score 2 and 3) from 92% in resting cells to 38% in sNTSR3 treated cells.

### Modification of the expression of proteins involved in cell-cell and cell-matrix junctions by sNTSR3

**Figure 4:**
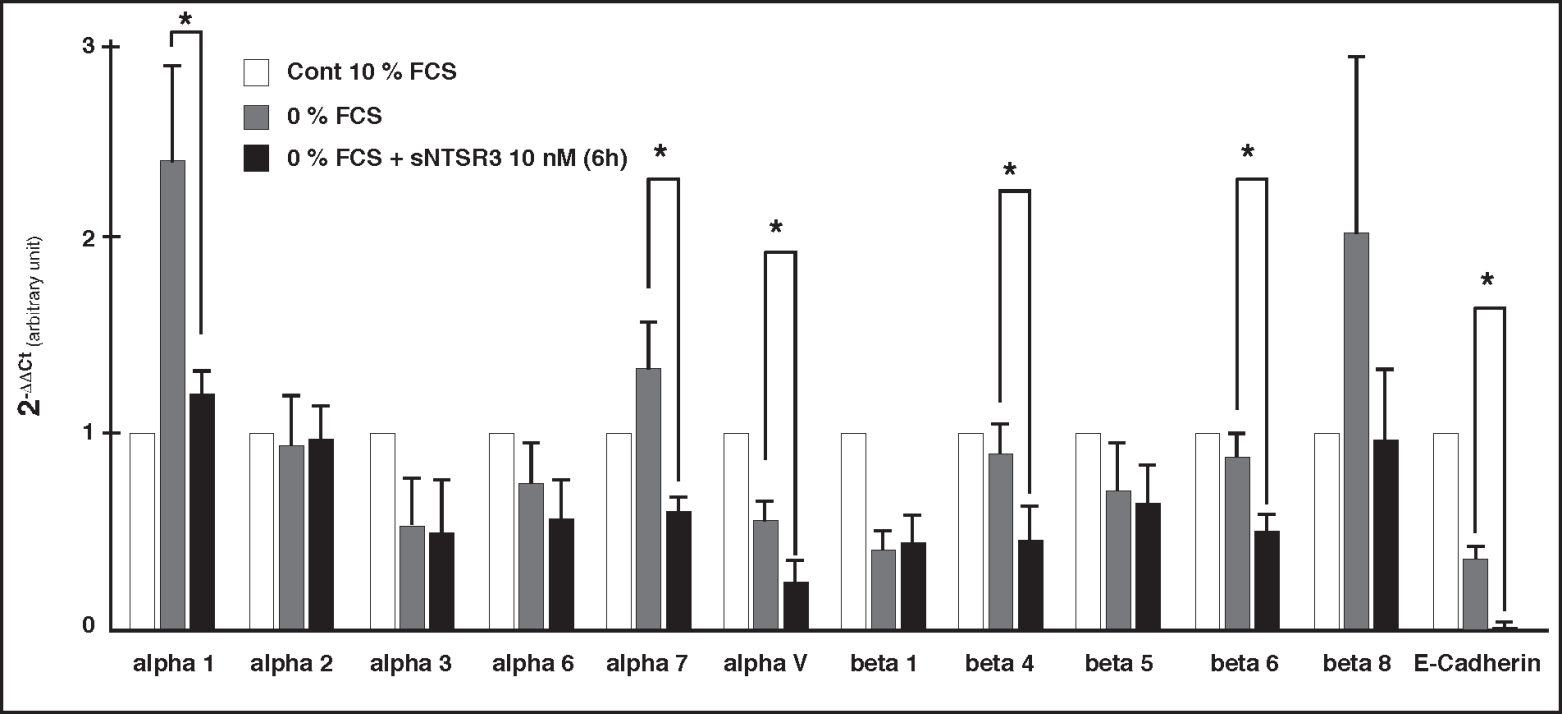
Expression profile of junction proteins Real-time pPCR was performed on the LightCycler™ 480 (Roche) using the LightCycler™ 480 SYBR Green 1 Master mix (Roche) using cyclophilin A and beta-actin as reference genes. Bar graph showing the subtype specific integrin and E-cadherin mRNA expression following 6h exposition to 0% FCS or 0% FCS + 10 nM sNTSR3 normalized with the control condition (10% FCS). Histograms are mean ± SEM from 4 independent determinations (* p<0.01).

The impressive changes observed in the HT29 cell shape, led us to investigate whether the expression of proteins involved in cell-cell junctions or cell adhesion were altered (Fig. 4). As suspected the mRNA expression of E-cadherin was almost totally inhibited after a 6 hours treatment with sNTSR3. This result confirms the observation of a modification of the cell-cell junction. Moreover the expression of several integrins, including alpha1, alpha7, alphaV, beta4, beta6 and beta8 was also inhibited following treatment of HT29 cells with sNTSR3 (Fig. 4). The integrins serve as adhesion receptor for extracellular matrix proteins, the decrease of their expression highlights a weakening of cell-matrix adhesion.

**Figure 5:**
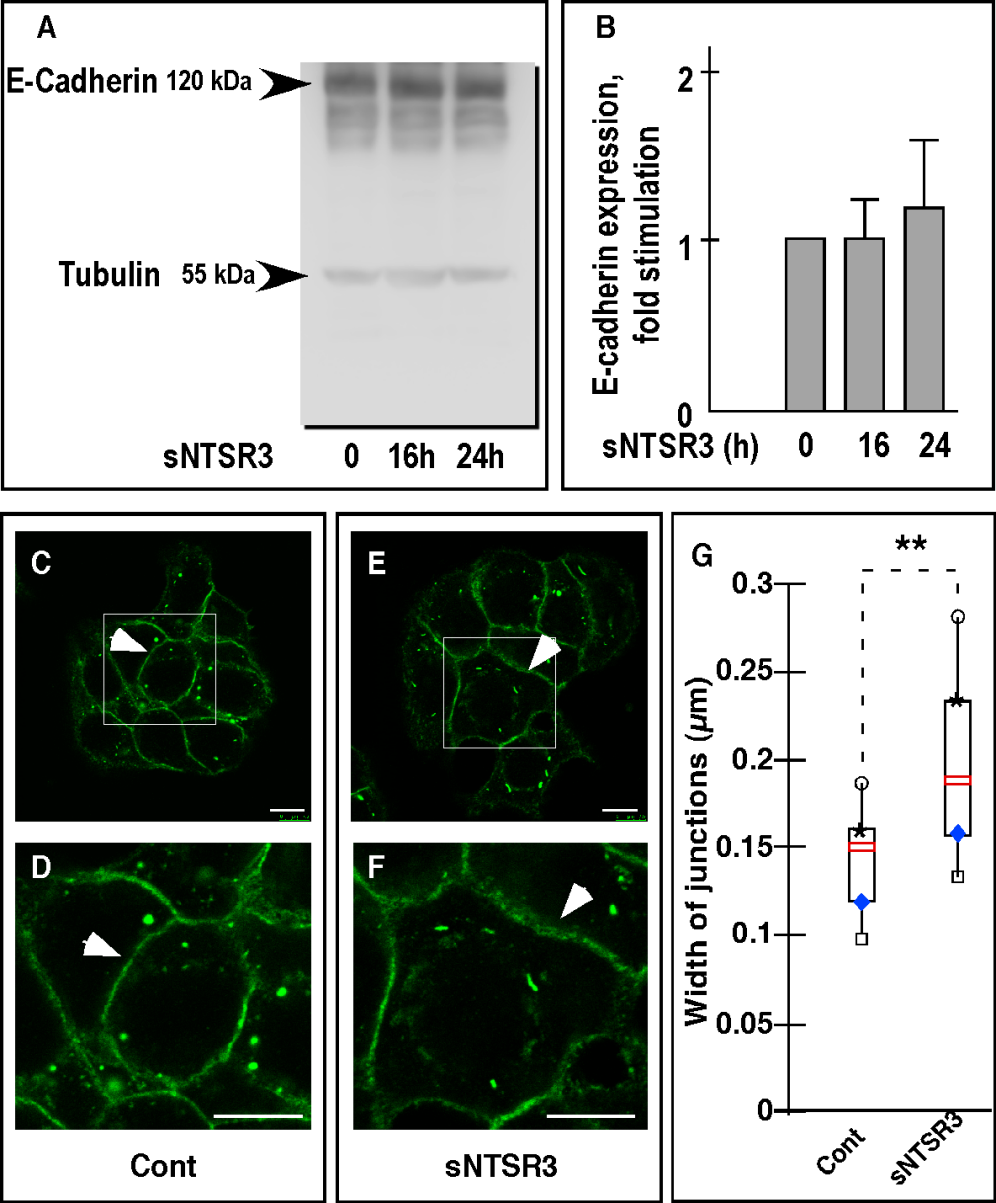
E-cadherin expression level in HT29 cells (A) Western blot analysis of the amount of E-cadherin expressed on HT29 cells treated or not with sNTSR3 for 16 and 24 hours. Total proteins extracted from HT29 cells cultivated under the conditions described above, were subjected to electrophoresis and the amount of E-cadherin was estimated using a specific anti-E-cadherin monoclonal antibody. (B) Quantification of E-cadherin expression was normalized using beta-tubulin as internal control. Histograms are mean ± SEM from three independent determinations. (C, D, E, F) Immunocytochemistry was performed using antibodies against E-Cadherin (green). Cells were incubated in the absence (C and D) or in the presence of sNTSR3 (10^−8^M) for 60 min in PBS buffer (E and F). Representative images are shown from 3 independent experiments. Scale bars : 10 μm. (G) The width of cell junctions was quantified by using ImageJ 1.4.3.67 software and expressed as median value obtained from 25 determinations, box plots show medians, 25% and 75% percentiles, and 5% and 95% percentiles. **p < 0.01.).

The protein content analysis of E-cadherin did not confirm the qPCR results (Fig. 5A and B). This is likely due to the high stability of E-cadherin during the time course of the experiment. The stability of the protein was confirmed by the absence of co-localization of E-cadherin labeling with lysosomes of sNTSR3 treated or not treated cells (not shown). However, the E-cadherin localization was disrupted by sNTSR3 treatment visualized by a more diffuse labeling of the protein of treated cells (Fig. 5E and F). The labeling obtained allowed us to observe an increase in the width of the E-cadherin labeling at the cell-cell junctions upon a 60 min treatment with sNTSR3 (arrows in Fig. 5E and F). Therefore, we measured the width of E-cadherin labeling and observed a significant increase from 0.15 to 0.19 μm (Fig. 5G) (n = 25, p<0.01), suggesting a disruption of cell-cell contacts.

These results indicate that the action of sNTSR3 means to fragilize the interactions between eptithelial cells, both at the cell-cell and the cell-matrix level.

### sNTSR3 promotes cancer cells detachment

**Figure 6:**
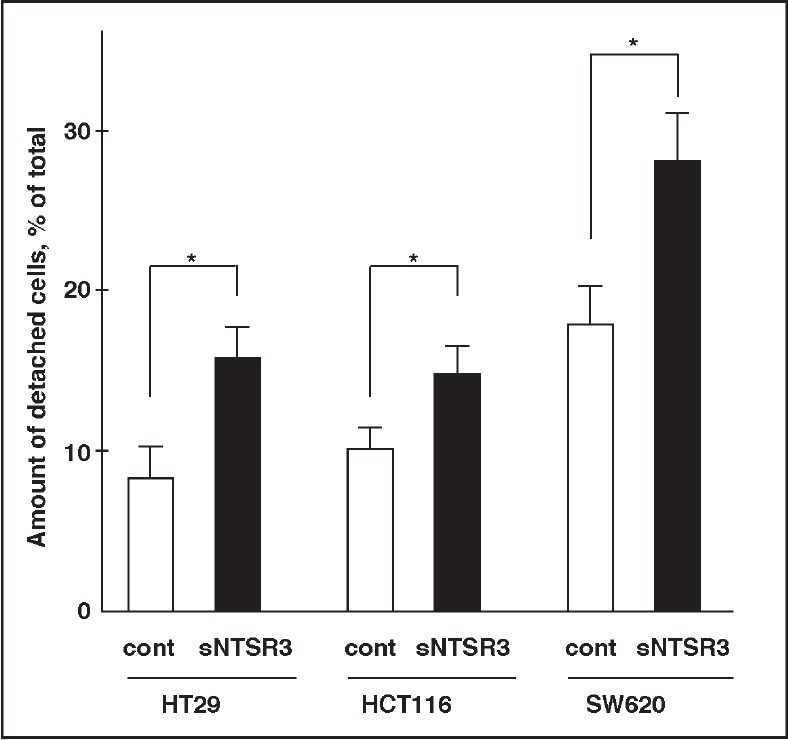
sNTSR3 increases cancer cells detachment

The weakening of cell-cell junctions and cell-matrix junctions could be the first step leading to cell detachment. To verify the potential role of sNTSR3 to trigger cell dissemination, we performed detachment experiments on both HT29, SW620 and HCT116 cell lines (three different human cancer cells). When cells were incubated under low serum concentration (0.1% FCS), the amount of detached cells after 24h was 8.3 ± 2.1% for HT29 cells, 10.1 ± 1.3% for HCT116 cells and 18.3± 2.4% for SW620 cells (Fig. 6). In the presence of 10^−8^M sNTSR3, this amount raised to 15.8 ± 2% for HT29 cells (p<0.05), to 14.9 ± 1.8% for HCT116 cells (p<0.05) and to 27.3 ± 3% for SW620 cells (p<0.05) (Fig. 6). These data suggest a role of sNTSR3 in cell detachment of colonic cancer cell lines.

HT29, HCT116 and SW620 cells were incubated in low serum concentration culture medium (0.1% FCS) in the absence (cont) or in the presence of 10^−8^M sNTSR3 for 24h at 37°C. Data represented the amount of detached cells normalized with the total amount of cells and are the mean ± SEM from 6 to 10 determinations. * p<0.05.

## DISCUSSION

NTSR3, also called sortilin, displays several known functions from a role as a receptor [20] to trigger the NT-induced human microglial migration, or a co-receptor as shown for its interaction with NTSR1 in HT29 cells to mediate NT signaling or with the p75 neurotrophin receptor (p75NTR) for proNGF-induced neuronal cell death [20,21,22]. NTSR3/sortilin also exerts intracellular functions to sort for examples the two-pore potassium channel TREK-1 to the plasma membrane [23], the sphingolipid activator proteins to lysosomes [24] and the brain-derived neurotrophic factor to the regulated secretion [25].

Interestingly, previous studies have already observed a correlation between NTSR3/sortilin expression and malignancy as characterized in glioma where the increase in the expression of NTSR3/sortilin was positively correlated with the malignancy of the tumor [26]. The level of NTSR3/sortilin expression was also increased in several colorectal cell lines that are models for primary and metastatic tumor cells [27]. These observations underline the putative function of NTSR3/sortilin in the process of tumoregenicity.

However, although the release by shedding of its extracellular domain upon activation of protein kinase C has been already described in primary cultured neurons and several cell lines including colonic cancer cells [8], nothing was known about the function of this released soluble protein. We recently showed that sNTSR3 is an active protein by its ability to bind, to internalize and to trigger intracellular signaling in HT29 cells. In the present work, we provide evidence that sNTSR3 strongly affects the HT29 cell morphology and adhesion properties.

First, we observed a modification of the geometric cell shape of cells treated with sNTSR3. The proportion of hexagons decreased in favour of pentagons. In agreement with these observations, a recent work describes a polygonal distribution favouring hexagons during cell-cell contact formation of human keratinocytes epithelial cells [16], a topology similar to those of Drosophila, Xenopus or Hydra [17,28]. A modification in the distribution of geometric cell shape indicates that the amount of junctions between cells are affected as shown by the depletion of Rock1/Rock2 kinases in human keratinocytes [16]. However, by contrast to human keratinocytes, the change of the geometric topology of HT29 cells is correlated with a change of cell surface of HT29 cells, a discrepancy certainly due to the difference of cell types.

The second important consequence of sNTSR3 treatment is a weakening of the cell-matrix contact. We observed a rapid and complete reorganization of the cytoskeleton of epithelial HT29 cells upon sNTSR3 stimulation. The actin microfilaments, surrounding the inner side of peripheral cells plasma membranes and responsible for the shape of the cell, are totally disorganized. Moreover, treated cells present an important increase in stress fibers, a response of the cell to modify the cell-matrix contact which can lead to migration. This observation is correlated with the activation of the focal adhesion kinase by sNTSR3 [12], and added to the weakening of extracellular junctions should lead to cell migration [29]. Since HT29 cells are non-migrating cells, these results suggest a first step of a new mechanism that could lead to cell detachment.

We demonstrated, by using a qPCR approach, that several alpha and beta integrins expressed in HT29 cells are down regulated after 6h exposition to sNTSR3. In particular, the expression of integrins alpha1, alpha7, alphaV, beta4, beta6 and beta8 is significantly decreased upon incubation with sNTSR3. Interestingly, the couple alphaV/beta8 has been shown to mediate epithelial homeostasis by regulating the activities of gene families involved in cell-matrix interactions [30]. The decrease of alphaV/beta8 expression induced by sNTSR3 could be responsible for the topological changes observed on HT29 cells. Loss of integrins couples has been detected in adenocarcinoma of the lung [31,32], in human colonic epithelial cells [33,34], and has been correlated with a poor prognosis. The decrease of several members of the integrin family induces a weakness of cell-matrix adhesion. As a result the cells tend to get loose from the plate. In order to compensate this phenomenon, we observed the apparition of actin stress fiber and focal adhesion point, which maintain the cell attached to the matrix.

Moereover, we demonstrated that the sNTSR3 treatment strongly affected cell-cell contact. As measured by qPCR analysis, the expression of E-cadherin, a transmembrane protein involved in cell-cell adhesion, decreased in sNTSR3 treated cells. Consequently we speculated that a loss of the protein will greatly reduce the strength of the interaction between cells. Even if we did not detect a decrease of the E-cadherin protein level, its cellular distribution was severely affected. At the cell junction, the localization of the protein appeared more diffuse. E-cadherin being part of the tight junction, it is present between the cells in the extracellular medium, and it maintains the neighbouring cells close to each other. We could speculate that the diffused E-cadherin localization at the cell-junction could be due to the separation of neighbouring cells, stretching the E-cadherin proteins and so increasing the width of the E-cadherin labelling. So the strength of the interaction between cells could be more fragile.

This hypothesis is strengthened by the observation of a disorganization of the desmosomes plaques, components of the tight junction. Electron microscopic experiments demonstrated that at the ultrastructural level both the number and the architecture of desmosomes were affected in sNTSR3 treated cells. The amount of strong intermediate filament bundles associated with the plaque densities which form desmosomes were dramatically reduced, as previously reported in keratinocytes depleted of a member of the plakin family, desmoplakin [19]. This disruption of desmosomes architecture is in agreement with the overall observations from this work where the action of sNTSR3 on HT29 cells is the embrittlement of cell-cell and cell-matrix interaction. The importance of desmosomes integrity has been demonstrated in several diseases and particularly in cancer progression. Indeed, the loss of desmosomes structure or components has been observed in the development or the progression of several human epithelial cancers (for reviews see [35,36]). Finally, we demonstrated that the weakening of cell-matrix and cell-cell contact lead to cell detachment: sNTSR3 treatment significantly increased both HT29 and HCT116 cell detachment (Fig. 6), an important property which could be responsible for further cell dissemination and metastasis.

In conclusion, our results indicate that sNTSR3 greatly modifies the HT29 cell shape, weaken the cell-cell interaction and reduces the cell-matrix interaction. sNTR3 may initiate the epithelial to mesenchymal transition, including desmosome structure disruption, cell-cell and cell-matrix weakening and then initiation of cell separation. sNTSR3, likely by acting through a specific membrane receptor which remains to be identified, is able to trigger an intracellular signaling (ie: Erk1/2 and/or Akt [12]) which leads to cellular modifications including a strong modification of cell adhesion and cell-cell contacts. Another possibility is that sNTSR3 can bind to specific growth factors and then can inhibit their effects. However, we recently demonstrated that sNTSR3 specifically binds to HT29 homogenates [12], which is rather in agreement with the first hypothesis. We clearly defined a role of sNTSR3 on tissue cohesion alteration with desmosome structure disruption and the initiation of cell separation. The importance of this finding on cell dissemination remains to be investigated.

**Table 1:** Primers targeting human integrins, E-cadherin and vimentin

Target	Forward primer (5'-3')	Reverse primer (5'-3')
Alpha1	GGTTCCTACTTTGGCAGTATT	AACCTTGTCTGATTGAGAGCA
Alpha2	GGAACGGGACTTTCGCAT	GGTACTTCGGCTTTCTCATCA
Alpha3	AAGGGACCTTCAGGTGCA	TGTAGCCGGTGATTTACCAT
Alpha6	TTGAATATACTGCTAACCCCG	TCGAAACTGAACTCTTGAGGATAG
Alpha7	CTGTTTCAGCTACATTGCAGTC	GCCTGGTGCTTGGGTTCT
AlphaV	AATCTTCCAATTGAGGATATCAC	AAAACAGCCAGTAGCAACAAT
Beta1	GAAGGGTTGCCCTCCAGA	GCTTGAGCTTCTCTGCTGTT
Beta4	AGACGAGATGTTCAGGGACC	GGTCTCCTCTGTGATTTGGAA
Beta5	GGAGCCAGAGTGTGGAAACA	GAAACTTTGCAAACTCCCTC
Beta6	TCAGCGTGACTGTGAATATCC	GTGACATTTGGAGCTGTTCAC
Beta8	AATTTGGTAGTGGAAGCCTATC	GTCACGTTTCTGCATCCTTC
Actin	GCTGTGCTACGTCGCCCTG	GGAGGAGCTGGAAGCAGCC
CycloA	CTCGAATAAGTTTGACTTGTGTTT	CTAGGCATGGGAGGGAACA
E-Cadherin	ACAGCCCCGCCTTATGATT	TCGGAACCGCTTCCTTCA
Vimentin	AAGAGAACTTTGCCGTTGAA	GTGATGCTGAGAAGTTTCGT

## METHODS

### Materials

Dulbecco's modified Eagle's medium (DMEM) was from Life Technologies Inc. and fetal calf serum from Lonza. Gentamicin, mowiol, paraformaldehyde, mammalian protease and phosphatase inhibitor cocktails were from Sigma France. The soluble recombinant human NTSR3/sortilin protein (Ser78-Asn755; MW = 76 kDa from R&D systems) was resuspended in PBS containing 0.1% bovine serum albumin (BSA) as a carrier following the manufacturer's recommendations. All control conditions were made using PBS containing 0.1% BSA. Monoclonal antibodies against E-cadherin and NTSR3/sortilin were from BD Bioscience. Alexa-594-phalloidin was from Invitrogen. Alexa-488 conjugated donkey anti-rabbit and Alexa-594 conjugated donkey anti-mouse antibodies were obtained from Jackson Immunoresearch Laboratories. HRP conjugated goat anti-rabbit and anti-mouse were from Cell Signalling.

### Cell culture

The human cell lines HT29, HCT116 and SW620 were maintained in DMEM supplemented with 10% FBS and 50 μg/ml gentamicin at 37°C under 5% CO_2_.

### Immunocytochemistry

Cells were plated on glass coverslips coated with 1 mg/ml poly-l-lysine. Two days later, cells were preincubated for 10 min in phosphate-buffered saline (PBS) and then either incubated or not (control) with sNTSR3 (10^−8^ M) for indicated times. Then, cells were fixed 20 min with 4% paraformaldehyde in PBS at room temperature. Coverslips were rinsed twice with PBS and incubated with 50 mM NH_4_Cl in PBS for 10 min to quench excess of free aldehyde groups. After 20 min in PBS containing 3% Horse Serum (HS) and 0.1% Triton-X100, cells were labelled with either the anti-E-cadherin (1/400) or with the Alexa-595-phalloidin (1/500) for 2 h at room temperature in PBS containing 0.5% HS and 0.1% Triton-X100. Cells were rinsed three times in PBS and incubated for 45 min at room temperature with a Alexa488-conjugated donkey anti-mouse antibody (1/400) in PBS containing 0.5% Horse Serum and 0.1% Triton-X100. After two washes with PBS and one with water, coverslips were mounted on glass slides with mowiol for confocal microscopy examination.

Confocal microscopy observations were performed with a Laser Scanning Confocal Microscope (TCS SP5, Leica, Rueil Malmaison, France) equipped with a DMI6000 inverted microscope, using a Plan Apo 63x/1,4 NA oil immersion objective. The fluorescent markers were respectively and sequentially excited by the 488 nm and 561 nm wavelengths of an argon and Diode Pumped Solid-State laser. Fluorescence was detected through 500-540 nm (Alexa-488) and 590-650 nm (Alexa-594) spectral windows. Images were acquired as single transcellular optical section and averaged over 12 scans/frame.

**Table 2:** Characterization of desmosomes in resting and sNTSR3 treated HT29 cells

Condition	Number^a^ Desmosomes/Slice	Intermediate filament associations^b^			
Desmosome score		0	1	2	3
- sNTSR3	5±0.34	2%	6%	31%	61%
+ sNTSR3	3.7±0.31*	31%	25%	23%	15%

a Average number of desmosomes per 70 nm cell slice, about 160-190 desmosomes were counted from 60 electronic views for each condition.

b Each desmosome was assigned to a score from 0-3, where 0 corresponds to no intermediate filaments visible near the desmosome, 1 corresponds to ambigous density near the intracellular plaque, 2 corresponds to a small number of intermediate filaments bundles, and 3 corresponds to strong intermediate filament bundles associated with the plaque. 189 desmosomes have been evaluated in control condition and 156 desmosomes have been evaluated in treated cells from 4 independent experiments.

* p<0.01 (Student's t-test)

For cell shape analysis (cell area and polygon class), images were analyzed using ImageJ software. For cell area analysis, intact cells in monolayer were numbered using nucleus labeling with a home-made macro. All the images of Dapi-labeled nuclei stored in a folder were treated automatically. First, the images were filtered to improve nucleus detection and second, the counting was done using the « Find Maxima » function configured to detect one local maxima per nucleus. The number of nuclei was normalized by the image surface. A total of 520 for control cells and 372 for sNTSR3-treated cells was determined. The polygon class of each cell was assigned by counting the number of vertices of the cell, only intact cells in monolayers were quantified (cells at the image border were excluded), a total of 252 for control cells and 265 for sNTSR3-treated cells from 3 independent experiments. The width of cell-cell junction in a monolayer was drawn and measured directly for each cell contact using ImageJ. A total of 55 cell contacts for control and 52 cell contacts for sNTSR3-treated cells were analyzed.

### Electron microscopy

For transmission electron microscopy analysis, the cells were fixed in 1.6% glutaraldehyde in 0.1 M phosphate buffer, rinsed in 0.1 M cacodylate buffer, post-fixed for 1h in 1% osmium tetroxide and 1% potassium ferrocyanide in 0.1 M cacodylate buffer to enhance the staining of membranes. Cells were then rinsed in distilled water, dehydrated in alcohols and lastly embedded in epoxy resin. Contrasted ultrathin sections (70 nm) were analyzed under a JEOL 1400 transmission electron microscope mounted with a Morada Olympus CCD camera.

### Western blot analysis

Proteins from HT29 cells incubated for various times with sNTSR3 (10^−8^ M) were denaturated by boiling at 95°C for 3 min using 2x Laemmli sample buffer, resolved using 10% acrylamide gels and subsequently electroblotted onto nitrocellulose membranes. Membranes were blocked with 5% skim milk in PBS and incubated in the same buffer with the mouse anti-E-cadherin antibody (1:1000) overnight at 4°C. The bound antibody was visualised using an HRP-conjugated goat anti-mouse antibodies by chemiluminescence reagents, with a Fujifilm Intelligente Darkbox LAS-3000 detection apparatus.

### Primers design and real-time qPCR

Primers were designed as previously described [13]. Primers, synthesized by Eurogentec, were targeted against human sequences of integrin alpha1, alpha2, alpha3, alpha6, alpha7, alphaV, beta1, beta4, beta5, beta6, beta7, beta8, and cyclophilin A and beta-actin as reference genes (Table 1). Every set of primers has been designed using intron spanning assay as required by the Universal Probe Library Software from Roche Diagnosis. Non specific products formation has been verified for each set of primers with the melting curve realized by the LightCycler™ 480 Software.

Real-time pPCR was performed on the LightCycler™ 480 (Roche) using the LightCycler™ 480 SYBR Green 1 Master mix (Roche). The PCR reaction was performed in 20 μl volume containing 16 ng cDNA, 10 μl 2x LightCycler™ 480 SYBR Green 1 Master mix and 1 μl of primer mix (10 μM forward primer, 10 μM reverse primer). The PCR profile was as follows: 5 min at 95°C, followed by 45 cycles of 10 sec at 95°C, 10 sec at 60°C and 10 sec at 72°C.

The Ct value of each gene of interest was normalized to the Ct of the reference genes as follows : ΔCT_norm_ = Ct_goi_-Ct_ref_ with Ct_ref_ = (Ct_bACT_ × Ct_CycloA_)^(1/2)^ with _norm_ = normalized, _goi_ = gene of interest, and _ref_ = reference gene. ΔΔCT = ΔCT experimental condition - ΔCT control condition. Values were expressed as 2^-ΔΔCt^ normalized using the 10% FCS condition as control.

### Cell detachment experiments

SW620, HCT116 and HT29 cells, plated in 48 well-dishes (4 × 10^5^ cells/dish), were incubated at low serum concentration (0.1%) for 24h in the absence (control condition) or in the presence of 10 nM sNTSR3. Cells recovered in the medium (detached cells) were quantified using a CASEY Model TT Cell Counter (Roche Applied Sciences, Indianapolis). Remaining plated cells were recovered by trypsin treatment and quantified as above.

### Statistics

Each median value is obtained from 6 to 12 determinations, box plots show medians, 25% and 75% percentiles, and 5% and 95% percentiles. Significance was determined using the Kruskal Wallis test: *p < 0.05 **p < 0.01 and ***p < 0.005, ns non-significant. For experiments with a low number of assays (n = 3 to 5 for cell shape, qPCR), the Mann and Whitney test was used : *p<0.01, ***p<0.005.

## References

[1] Reubi JC (2003). Peptide receptors as molecular targets for cancer diagnosis and therapy. Endocr Rev.

[2] Pini A, Falciani C, Bracci L (2008). Branched peptides as therapeutics. Curr Protein Pept Sci.

[3] Thiery JP (2002). Epithelial-mesenchymal transitions in tumour progression. Nat Rev Cancer.

[4] Kasina S, Scherle PA, Hall CL, Macoska JA (2009). ADAM-mediated amphiregulin shedding and EGFR transactivation. Cell Prolif.

[5] Marcusson EG, Horazdovsky BF, Cereghino JL, Gharakhanian E, Emr SD (1994). The sorting receptor for yeast vacuolar carboxypeptidase Y is encoded by the VPS10 gene. Cell.

[6] Hermey G (2009). The Vps10p-domain receptor family. Cell Mol Life Sci.

[7] Hermey G, Sjogaard SS, Petersen CM, Nykjaer A, Gliemann J (2006). Tumour necrosis factor alpha-converting enzyme mediates ectodomain shedding of Vps10p-domain receptor family members. Biochem J.

[8] Navarro V, Vincent JP, Mazella J (2002). Shedding of the luminal domain of the neurotensin receptor-3/sortilin in the HT29 cell line. Biochem Biophys Res Commun.

[9] Willnow TE, Petersen CM, Nykjaer A (2008). VPS10P-domain receptors - regulators of neuronal viability and function. Nat Rev Neurosci.

[10] Petersen CM, Nielsen MS, Nykjaer A, Jacobsen L, Tommerup N (1997) Molecular identification of a novel candidate sorting receptor purified from human brain by receptor-associated protein affinity chromatography. J Biol Chem.

[11] Mazella J, Zsurger N, Navarro V, Chabry J, Kaghad M (1998) The 100-kDa neurotensin receptor is gp95/sortilin, a non-G-protein-coupled receptor. J Biol Chem.

[12] Massa F, Devader C, Beraud-Dufour S, Brau F, Coppola T (2013) Focal adhesion kinase dependent activation of the PI3 kinase pathway by the functional soluble form of neurotensin receptor-3 in HT29 cells. Int J Biochem Cell Biol.

[13] Dingemans AM, van den Boogaart V, Vosse BA, van Suylen RJ, Griffioen AW (2010) Integrin expression profiling identifies integrin alpha5 and beta1 as prognostic factors in early stage non-small cell lung cancer. Mol Cancer.

[14] Jen CJ, Chen HI, Lai KC, Usami S (1996). Changes in cytosolic calcium concentrations and cell morphology in single platelets adhered to fibrinogen-coated surface under flow. Blood.

[15] Lishner M, Zismanov V, Tohami T, Tartakover-Matalon S, Elis A (2008) Tetraspanins affect myeloma cell fate via Akt signaling and FoxO activation. Cell Signal.

[16] Kalaji R, Wheeler AP, Erasmus JC, Lee SY, Endres RG (2012) ROCK1 and ROCK2 regulate epithelial polarisation and geometric cell shape. Biol Cell.

[17] Farhadifar R, Roper JC, Aigouy B, Eaton S, Julicher F (2007). The influence of cell mechanics, cell-cell interactions, and proliferation on epithelial packing. Curr Biol.

[18] Green KJ, Gaudry CA (2000). Are desmosomes more than tethers for intermediate filaments?. Nat Rev Mol Cell Biol.

[19] Acehan D, Petzold C, Gumper I, Sabatini DD, Muller EJ (2008) Plakoglobin is required for effective intermediate filament anchorage to desmosomes. J Invest Dermatol.

[20] Martin S, Vincent JP, Mazella J (2003). Involvement of the neurotensin receptor-3 in the neurotensin-induced migration of human microglia. J Neurosci.

[21] Nykjaer A, Lee R, Teng KK, Jansen P, Madsen P (2004) Sortilin is essential for proNGF-induced neuronal cell death. Nature.

[22] Martin S, Navarro V, Vincent JP, Mazella J (2002). Neurotensin receptor-1 and -3 complex modulates the cellular signaling of neurotensin in the HT29 cell line. Gastroenterology.

[23] Mazella J, Petrault O, Lucas G, Deval E, Beraud-Dufour S (2010) Spadin, a sortilin-derived peptide, targeting rodent TREK-1 channels: a new concept in the antidepressant drug design. PLoS Biol.

[24] Lefrancois S, Zeng J, Hassan AJ, Canuel M, Morales CR (2003). The lysosomal trafficking of sphingolipid activator proteins (SAPs) is mediated by sortilin. EMBO J.

[25] Chen ZY, Ieraci A, Teng H, Dall H, Meng CX (2005) Sortilin controls intracellular sorting of brain-derived neurotrophic factor to the regulated secretory pathway. J Neurosci.

[26] Xiong J, Zhou L, Yang M, Lim Y, Zhu YH (2013) ProBDNF and its receptors are upregulated in glioma and inhibit the growth of glioma cells in vitro. Neuro Oncol.

[27] Akil H, Perraud A, Melin C, Jauberteau MO, Mathonnet M (2011). Fine-tuning roles of endogenous brain-derived neurotrophic factor, TrkB and sortilin in colorectal cancer cell survival. PLoS One.

[28] Gibson MC, Patel AB, Nagpal R, Perrimon N (2006). The emergence of geometric order in proliferating metazoan epithelia. Nature.

[29] Yuan SY, Shen Q, Rigor RR, Wu MH (2012). Neutrophil transmigration, focal adhesion kinase and endothelial barrier function. Microvasc Res.

[30] Mu D, Cambier S, Fjellbirkeland L, Baron JL, Munger JS (2002) The integrin alpha(v)beta8 mediates epithelial homeostasis through MT1-MMP-dependent activation of TGF-beta1. J Cell Biol.

[31] Adachi M, Taki T, Huang C, Higashiyama M, Doi O (1998) Reduced integrin alpha3 expression as a factor of poor prognosis of patients with adenocarcinoma of the lung. J Clin Oncol.

[32] Takenaka K, Shibuya M, Takeda Y, Hibino S, Gemma A (2000) Altered expression and function of beta1 integrins in a highly metastatic human lung adenocarcinoma cell line. Int J Oncol.

[33] Koretz K, Schlag P, Boumsell L, Moller P (1991). Expression of VLA-alpha 2, VLA-alpha 6, and VLA-beta 1 chains in normal mucosa and adenomas of the colon, and in colon carcinomas and their liver metastases. Am J Pathol.

[34] Stallmach A, von Lampe B, Matthes H, Bornhoft G, Riecken EO (1992). Diminished expression of integrin adhesion molecules on human colonic epithelial cells during the benign to malign tumour transformation. Gut.

[35] Dusek RL, Attardi LD (2011). Desmosomes: new perpetrators in tumour suppression. Nat Rev Cancer.

[36] Brooke MA, Nitoiu D, Kelsell DP (2012). Cell-cell connectivity: desmosomes and disease. J Pathol.

